# Molluscicidal effectiveness of Luo-Wei, a novel plant-derived molluscicide, against *Oncomelania hupensis*, *Biomphalaria alexandrina* and *Bulinus truncatus*

**DOI:** 10.1186/s40249-019-0535-7

**Published:** 2019-03-31

**Authors:** Tie-Wu Jia, Wei Wang, Le-Ping Sun, Shan Lv, Kun Yang, Neng-Min Zhang, Xi-Bao Huang, Jian-Bing Liu, Han-Cheng Liu, Rui-Hua Liu, Fathia A. Gawish, Mohamed R. Habib, Mohamed A. El-Emam, Charles H. King, Xiao-Nong Zhou

**Affiliations:** 1 National Institute of Parasitic Diseases, Chinese Center for Disease Control and Prevention, Shanghai, 200025 China; 2 Chinese Center for Tropical Diseases Research, Shanghai, 200025 China; 3 WHO Collaborating Centre for Tropical Diseases, Shanghai, 200025 China; 4 National Center for International Research on Tropical Diseases, Ministry of Science and Technology, Shanghai, 200025 China; 5 Key Laboratory of Parasite and Vector Biology, Ministry of Health, Shanghai, 200025 China; 6 Communicable Diseases Cluster, World Health Organization Regional Office for Africa (WHO/AFRO), PO Box 06, Brazzaville, Congo; 7 Key Laboratory of National Health Commission on Parasitic Disease Control and Prevention, Jiangsu Provincial Key Laboratory on Parasites and Vector Control Technology, Jiangsu Institute of Parasitic Diseases, Wuxi, 214064 China; 8 Hubei Jinhaichao Science & Technology Co., Ltd, Wuhan, 430206 China; 9 Hubei Provincial Center for Disease Control and Prevention, Wuhan, 430079 China; 10 School of Chemistry and Chemical Engineering, Wuhan Textile University, Wuhan, 430200 China; 11 Department of Medical Malacology, Theodor Bilharz Research Institute (TBRI), Imbaba, Giza, 12411 Egypt; 12 Center for Global Health and Diseases, Case Western Reserve University, Cleveland, OH USA; 13 Schistosomiasis Consortium for Operational Research and Evaluation, University of Georgia, Athens, GA USA

**Keywords:** Schistosomiasis, Luo-Wei, Plant-derived molluscicide, *Oncomelania hupensis*, *Biomphalaria alexandrina*, *Bulinus truncatus*, Molluscicidal activity

## Abstract

**Background:**

Control of snail intermediate hosts has been proved to be a fast and efficient approach for interrupting the transmission of schistosomiasis. Some plant extracts have shown obvious molluscicidal activity, and a new compound Luo-Wei, also named tea-seed distilled saponin (TDS), was developed based on the saponins extracted from *Camellia oleifera* seeds. We aimed to test the molluscicidal activity of 4% TDS against the intermediate host snails in China and Egypt, and evaluate its environmental safety to non-target organisms.

**Methods:**

In the laboratory, *Oncomelania hupensis*, *Biomphalaria alexandrina* and *Bulinus truncatus* were exposed to 4% TDS, and the median lethal concentration (LC_50_) was estimated at 24, 48 and 72 h. In the field, snail mortalities were assessed 1, 2, 3 and 7 d post-immersion with 2.5 g/m^3^ 4% TDS and 1, 3, 7 and 15 d post-spraying with 5 g/m^2^ 4% TDS. In addition, the acute toxicity of 4% TDS to Japanese quail (*Coturnix japonica*), zebrafish (*Brachydanio rerio*) and freshwater shrimp (*Macrobrachium nipponense*) was assessed by estimations of LC_50_ or median lethal dose (LD_50_).

**Results:**

In the laboratory, the LC_50_ values of 4% TDS for *O. hupensis* were 0.701, 0.371 and 0.33 mg/L at 24, 48 and 72 h, respectively, and 4% TDS showed a 0.33 mg/L 24 h LC_50_ against *B. alexandrina*, and a 1.396 mg/L 24 h LC_50_ against *B. truncatus*. Across all study regions, the pooled mortalities of *O. hupensis* were 72, 86, 94 and 98% at 1, 2, 3 and 7 d, following field immersion of 4% TDS at a dose of 2.5 g/m^3^, and were 69, 77, 85 and 88% at 1, 3, 7 and 15 d, following field spraying at 5 g/m^2^, respectively. 4% TDS had moderate toxicity to Japanese quail (7 d LD_50_ > 60 mg/kg) and to shrimp (96 h LC_50_ = 6.28 mg/L; 95% *CI*: 3.53–11.2 mg/L), whereas its toxicity to zebrafish was high (96 h LC_50_ = 0.15 mg/L; 95% *CI*: 0.14–0.17 mg/L).

**Conclusions:**

4% TDS is active against *O. hupensis*, *B. alexandrina* and *B. truncatus* under laboratory and field conditions, and it may be a candidate molluscicide of plant origin.

**Electronic supplementary material:**

The online version of this article (10.1186/s40249-019-0535-7) contains supplementary material, which is available to authorized users.

## Multilingual abstracts

Please see Additional file 1 for translations of the abstract into the five official working languages of the United Nations.

## Background

Schistosomiasis is one of the most widespread parasitic infections and the second most prevalent parasitic disease in the world in terms of overall morbidity, socioeconomic and public health importance [1]. The three major species of schistosomes that infect humans, including *Schistosoma japonicum*, *S. mansoni* and *S. haematobium*, are transmitted by specific genera of snails, i.e., *Oncomelania* spp*.*, *Biomphalaria* spp*.*, and *Bulinus* spp*.*, respectively [1]. Parasitic *Schistosoma* infections in humans depend absolutely on the local presence of their intermediate freshwater snail hosts [1]. Molluscicide-based control of snail intermediate hosts is a fast and efficient approach for interrupting the transmission of this parasite [2, 3]. Niclosamide has been recommended by the World Health Organization (WHO) as a molluscicide since the 1960s and is still the molluscicide of choice [4]. However, the synthetic chemical molluscicides typically used to control these snails are expensive and can be toxic to other living organisms in the snail environmental habitat. Recent phytochemical screening has indicated that many plants are endowed with pesticidal properties that can be harnessed cheaply for vector control [5], and plant extracts have been studied as alternatives to chemical molluscicides [6, 7].

**Fig. 1:**
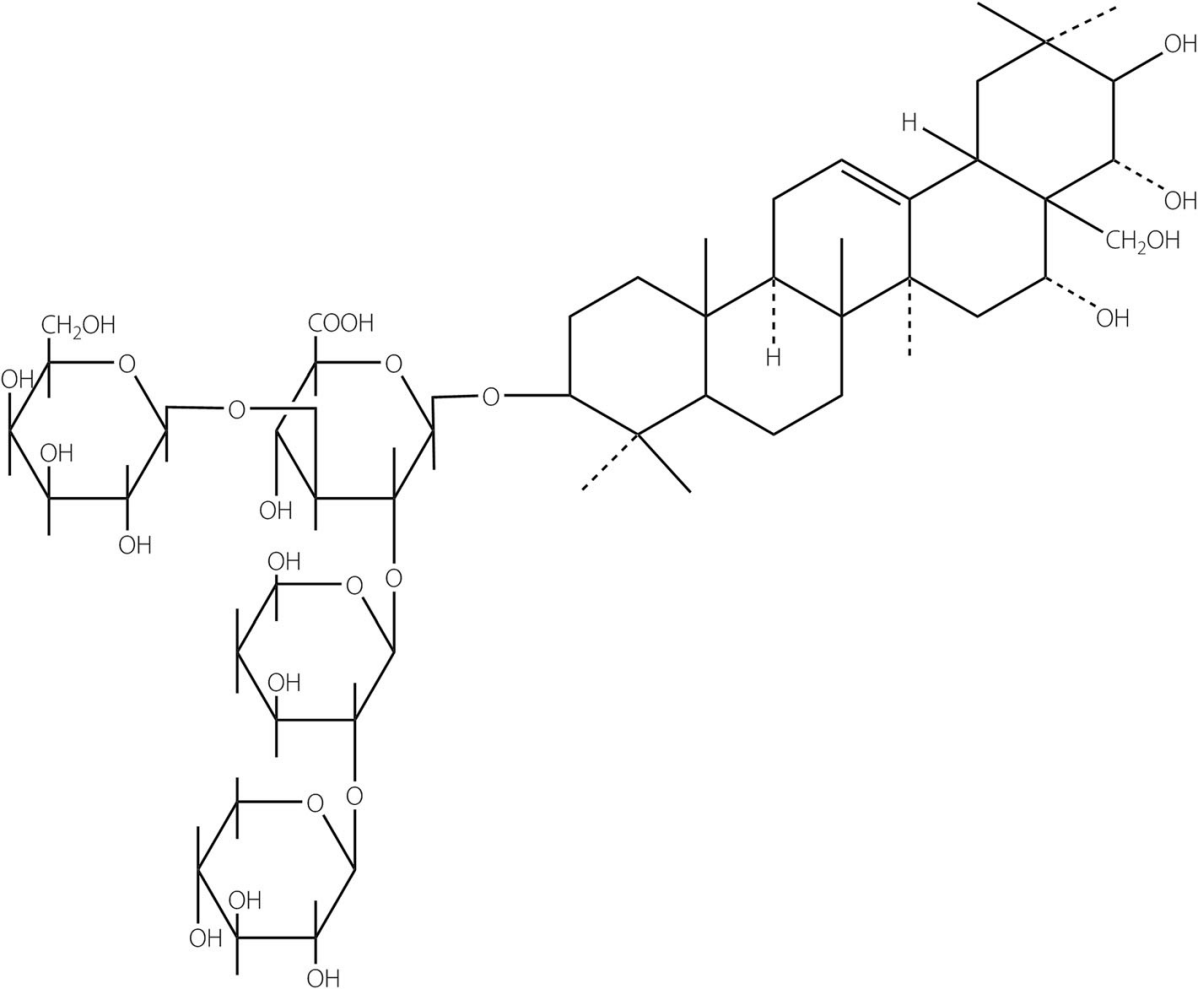
Structural formula of TDS (Luo-Wei) and its chemical name. TDS (C_52_H_84_O_24_, molecular weight 1093.23) is a pentacyclic triterpenoid saponin extracted from the tea seed pomace (*Camellia oleifera*) that is left after commercial pressing of seeds for tea oil. Its chemical name listed at the International Union of Pure and Applied Chemistry (IUPAC) is(3β, 16α)-28-oxo-D-xylopyranose-(1 → 3)-O -β-D-pyran-(1 → 4)-O-6-deoxy-α-L-mannopyranosyl-(1 → 2)-β-D-xylopyranose-17 -hydroxymethyl-16, 21, 22-trihydroxyoleanolic-12-alkene

A new molluscicide has been discovered from an alternative botanical source, *Camellia oleifera,* which is widely cultivated in South China. The seeds of this plant can be pressed to yield edible tea oil (camellia oil), and its byproduct, tea seed pomace, is normally discarded as waste or is used as a natural detergent or organic fertilizer with limited economic value. However, there are about 8% crude saponins in tea seed pomace that show significant biological and pharmacological activities [8, 9]. During the 2000s, a new compound was developed by alkaline hydrolysis and purification of the saponins extracted from the tea seed pomace, termed tea-seed distilled saponin (TDS) [10, 11], for which the registered chemical name at the International Union of Pure and Applied Chemistry (IUPAC) is (3β, 16α)-28-oxo-D -xylopyranose-(1 → 3)-O-β-D-pyran-(1 → 4)-O-6-deoxy-α-L-mannopyranosyl-(1 → 2)- β-D-xylopyranose-17-hydroxymethyl-16,21,22-trihydroxyoleanolic-12-alkene (C_52_H_84_O_24_, MW 1093.23; Fig. 1). As pentacyclic triterpenoid saponins (PTSs), the pretest showed that the technical material of TDS (91.6%) was active against *O. hupensis* snails (Additional file 2: Table S1). Listed as a new plant-derived molluscicide, its common name was registered as Luo-Wei (which means “snail threatener” in Chinese) by the Ministry of Agriculture (MoA) of People’s Republic of China in 2007, and the 4% powder formulation of TDS (4% TDS) was approved for pesticide use in China by the MoA in 2008. In the present report, we aimed to assess the molluscicidal activity of 4% TDS against the intermediate host snails of schistosomes in the laboratory and the field, and evaluate the environmental safety of 4% TDS to non-target organisms.

## Methods

### Laboratory molluscicidal activity test

To test the molluscicidal activity of 4% TDS against *O. hupensis* in the laboratory, *O. hupensis* snails were collected from the marshland in Yangzhou City, Jiangsu Province along the Yangtze River basin, and were given indoor feeding for 1 week before testing. Active and mature snails were selected for testing, and 4% TDS were prepared to give 9 concentrations of 0.04, 0.08, 0.16, 0.31, 0.63, 1.25, 2.5, 5 and 10 mg/L in the dechlorinated tap water. Then, 10 snails were exposed to each concentration for 24, 48 and 72 h at a room temperature of 25 ± 1 °C, respectively, and snails immersed in dechlorinated tap water served as controls. They were rinsed with dechlorinated water and incubated for a further 48 h to determine whether they were dead or alive.

To test the activity of 4% TDS against *B. alexandrina* and *B. truncatus* snails in the laboratory, these snail species were collected from water bodies in Giza governorate, Egypt, transferred to the laboratory, washed, and examined for natural trematode infections. Healthy non-infected snails were maintained at the Department of Medical Malacology, Theodor Bilharz Research Institute (TBRI), Egypt, in plastic aquaria provided with dechlorinated tap water (10 snails/L, 25 ± 1 °C) for at least 3 weeks before tests, and 4% TDS were formulated to concentrations of 0.75, 1, 1.25, 1.5, 1.75, 2, 2.25, 2.5, 2.75 mg/L in dechlorinated tap water. Ten snails were exposed to each concentration for 24, 48 and 72 h at a room temperature of 25 ± 1 °C, respectively, and snails immersed in dechlorinated tap water served as controls. They were rinsed with dechlorinated water and incubated for a further 48 h to determine whether they were dead or alive. All tests were repeated in triplicate, and the median lethal concentration (LC_50_) was calculated [12].

### Field assessment of molluscicidal activity

**Fig. 2:**
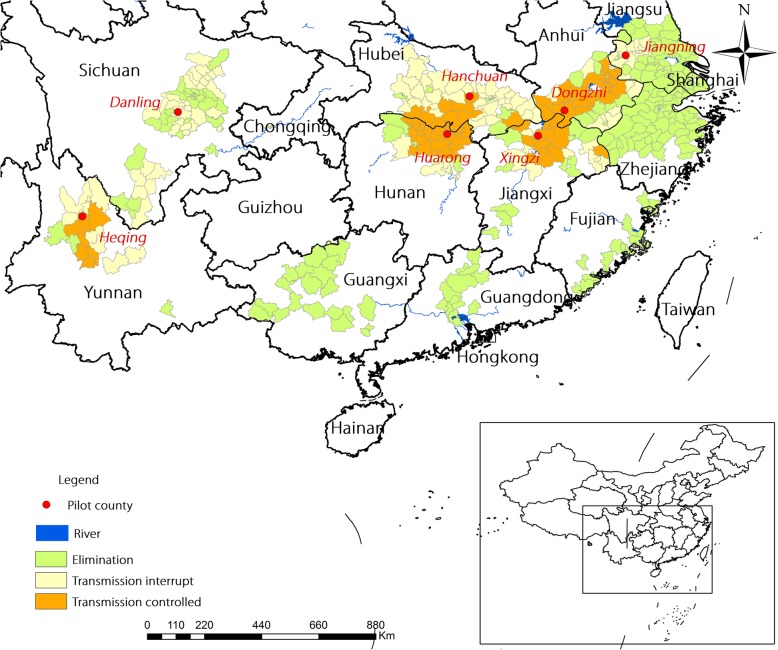
Distribution of the study areas to test the field molluscicidal efficacy of 4% TDS against *Oncomelania hupensis* in China**.** During the period between 2011 and 2014, field assessment of 4% TDS against *O. hupensis* was conducted in provinces that were endemic for *S. japonicum* in China, including Hunan, Hubei, Jiangxi, Anhui, Jiangsu, Sichuan, and Yunnan

During the period between 2011 and 2014, field assessment of 4% TDS against *O. hupensis* was conducted in provinces that were endemic for *S. japonicum* in China, including Hunan, Hubei, Jiangxi, Anhui, Jiangsu, Sichuan, and Yunnan (Fig. 2). Snail-inhabited ditches and plots with densities of living snails > 10 snails per 0.1 m^2^ (33 cm × 33 cm) were selected for immersion and spraying tests, respectively. The recommended temperature for application was 18–35 °C and relative humidity 50–80% in the field, without heavy rain. The field experiment was repeated if the snail mortality was more than 10% in the blank control group [13].

In the immersion test, the selected irrigation ditches were separated by non-water partitions (> 1 m), and each section was more than 40 m of length with at least 30 m^3^ of water volume. The upper section was for the water-only control group and the lower section was for the 4% TDS or 50% wettable powder of niclosamide ethanolamine salt (WPN; Nantong Luosen Chemical Co. Ltd., Nantong, China) treatment group. The dose concentrations tested were 2.5 g/m^3^ for 4% TDS and 2 g/m^3^ for 50% WPN. Each ditch section was topped up to test volume by pumping water to maintain water level after the vegetation clearance. The active mature snails from the same field were selected and packed into 18 nylon bags (50 snails/bag). In each treatment group, three monitoring sites were set equidistantly with each site of three bags of snails. A bag was taken from each site for mortality observation 1, 2, 3 and 7 d post-immersion.

In the spraying test, a flat plot of snail habitats was selected in the lake marshland, river floodplain, or mountain terrace and divided into three sections, one for the 4% TDS group (≥ 3000 m^2^), one for 50% WPN(≥ 3000 m^2^), and the other for the water-only control area (≥ 600 m^2^), with a spacing distance of > 10 m between groups. The vegetation was cut to under 10 cm and removed before spraying. The dose concentrations were 5 g/m^2^ for 4% TDS and 2 g/m^2^ for 50% WPN. Snail mortalities were observed 1, 3, 7 and 15 d post-spraying.

### Ecotoxicological test of 4% TDS

Environmental safety assessment of 4% TDS for non-target organisms, including birds, fish, and aquatic invertebrates, were conducted according to the *Test Guidelines on Environmental Safety Assessment for Chemical Pesticides* [14]. Japanese quail (*Coturnix japonica*), zebrafish (*Brachydanio rerio*) and freshwater shrimp (*Macrobrachium nipponense*) were selected as representative Chinese animals for these acute toxicity tests. A single dose gavage method was used to evaluate the acute toxicity of 4% TDS to Japanese quail. Five treatment groups (including five dose levels of 6, 12, 24, 36, and 60 mg/kg body weight of 4% TDS) with a blank control were applied, of which each group consisted of 10 birds (5 males and 5 females). After dosing, toxic signs and mortalities were continuously observed and recorded at 1, 2, 3, and 7 d. In the test of acute aquatic toxicity, 10 freshwater fish or shrimp were used at each test concentration and in the controls (0, 0.04, 0.10, 0.15, 0.20 and 0.40 mg/L in zebrafish and 0, 1, 2, 3, 5 and 10 mg/L in shrimps, respectively), complying with the semi-static procedure (renewal of the test solution every 24 h). The fish or shrimp were exposed to the test substance for a period of 96 h. Mortalities were recorded at 24, 48, 72 and 96 h and the LC_50_ or the median lethal dose (LD_50_) values were calculated [12].

### Statistical analysis

All LC_50_ or LD_50_ values and their 95% confidence intervals (*CI*s), probit/log concentration regression equations, and slope were calculated using the Bliss’s probit method with the computer program PoloPlus version 1.0 (LeOra Software; Petaluma, CA, USA) [12, 15]. Parameters for data files analyzed by PoloPlus were as follows: probit model, concentrations converted to logarithms, and no natural response [15]. The parallel and equal hypothesis tests of probit mortality lines were done and the 95% *CI* of lethal concentration ratios (LCRs) were calculated to compare the susceptibility of different snail species to 4% TDS [16]. If the 95% *CI* of LCR included 1, the LCRs were not considered significantly different.

A chi-square test was used to examine the differences of mortalities between time points after immersing and spraying. Open Meta-analyst software (Brown University; Providence, RI, USA) was used to make a pooled estimate of snail mortality and compare the difference of molluscicidal activity against *O. hupensis* snails between 4% TDS and 50% WPN using relative risk (RR) calculation [17]. Significance of this comparison was determined only if 1 was not included in the 95% *CI* of the observed RR. The likelihood ratio (LR) and LCR at 50% response level were calculated, and a *P* value < 0.05 was considered statistically significant.

## Results

### Laboratory molluscicidal activity

**Fig. 3:**
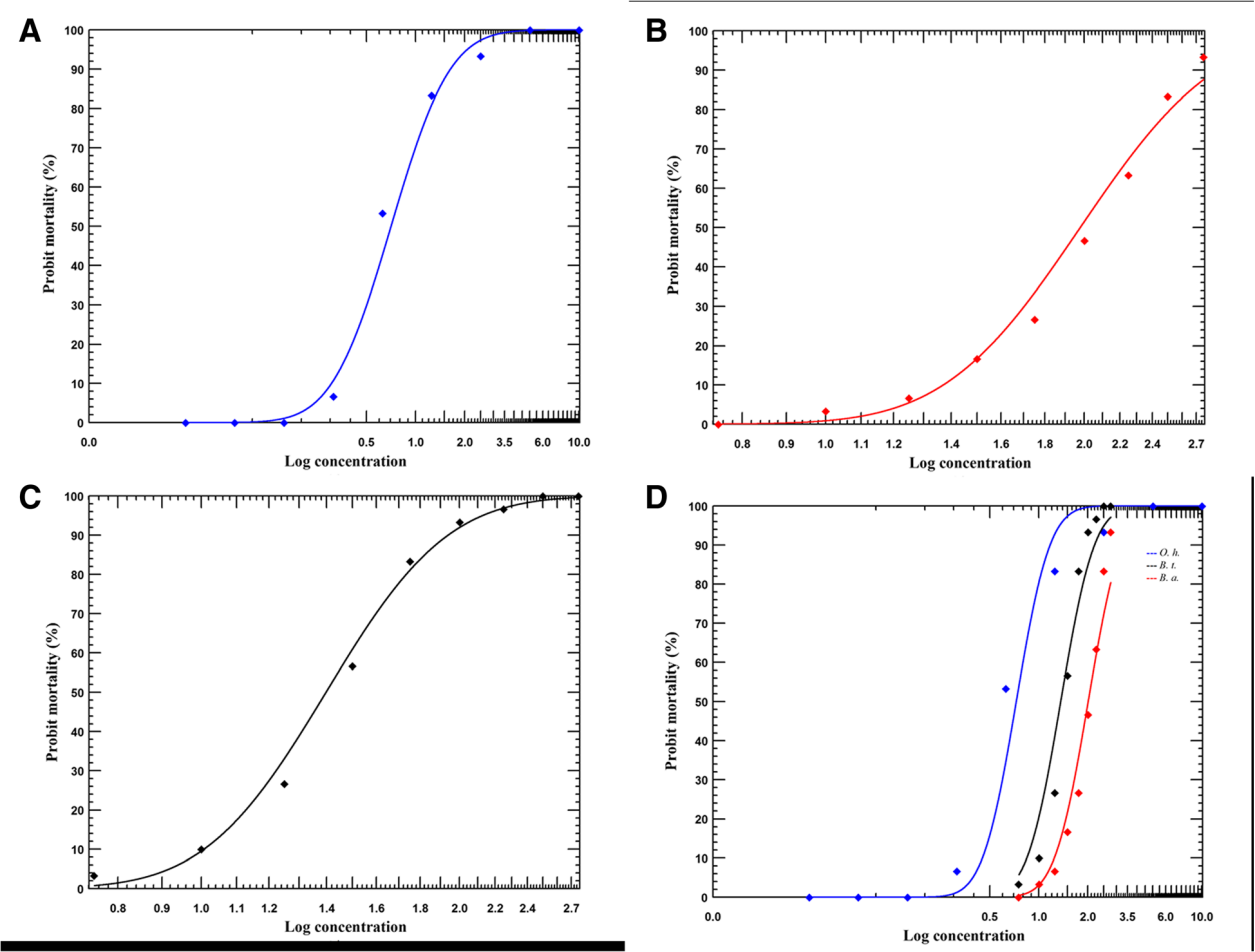
Dose-response curves of *Oncomelania hupensis*, *Biomphalaria alexandrina,* and *Bulinus truncatus* snails subjected to aqueous dilutions of 4% TDS for 24 h. **a** *Oncomelania hupensis*; **b** *Biomphalaria alexandrina*; **c** *Bulinus truncatus*; **d** The equality and parallelism of the regression lines of different snail species after 24 h exposure, tested by LR. *O. h., Oncomelania hupensis; B. a., Biomphalaria alexandrina; B. t., Bulinus truncatus.* The equality of the regression lines were tested using the likelihood ratio (LR). In general, there are significant differences between slopes and intercepts of lines (*χ*^2^ = 206, *P* < 0.05). The parallelism of slopes is tested by the LR. In general, there are significant differences between the regression lines (*χ*^2^ = 39.6, *P* < 0.05)

In the laboratory, the LC_50_ values for 4% TDS with their respective 95% *CI*s and slopes for each snail populations are shown in Table 1 and Fig. 3a–c. The LC_50_ values differed significantly among snail species and depending on treatment duration (Table 1 and Additional file 2: Table S2 and S3). With longer duration of exposure, the LC_50_ values of 4% TDS against *O. hupensis* decreased from 0.701 (0.581–0.842) mg/L at 24 h, to 0.371 (0.315–0.436) mg/L at 48 h, and to 0.33 (0.284–0.385) mg/L at 72 h, respectively. The log concentration-probit mortality curves for 24 h exposure were significantly different among snail species (the equality tested by LR: *χ*^2^ = 206, *P* < 0.05; the parallelism by LR: *χ*^2^ = 39.57, *P* < 0.05) (Fig. 3d). The lowest lethal concentration of snail populations at 24 h exposure was observed for *O. hupensis*, for which LC_50_ was nearly three-fold lower than that of *B. alexandrina* (1.975 mg/L) or *B. truncatus* (1.396 mg/L), with observed LCR_50_ (*O. hupensis*/*B. alexandrina/B. truncatus*) of 0.355 (95% *CI*: 0.293–0.43) and 0.502 (95% *CI*: 0.414–0.609), respectively (Table 1).

**Table 1:** Molluscicidal activity of 4% TDS against *Oncomelania hupensis*, *Biomphalaria alexandrina* and *Bulinus truncatus* by the immersion test in the laboratory

Snail species	Time (h)	*N* ^a^	*n*	Slope ± SE	*χ* ^2 b^	LC_50_ (95% *CI*)	LCR_50_ (95% *CI*)^c^
*Oncomelania hupensis*	24	9	270	3.522 ± 0.445	4.290	0.701 (0.581, 0.842)	Reference
	48	9	270	4.730 ± 0.675	5.073	0.371 (0.315, 0.436)	1.891 (1.484, 2.411)
	72	9	270	5.514 ± 0.868	2.097	0.330 (0.284, 0.385)	2.125 (1.680, 2.687)
*Biomphalaria alexandrina*	24	9	270	8.043 ± 0.910	4.576	1.975 (1.868, 2.092)	0.355 (0.293, 0.430)
*Bulinus truncatus*	24	9	270	9.014 ± 0.929	4.154	1.396 (1.312, 1.477)	0.502 (0.414, 0.609)

^a^*N*, number of dose groups (excluding control); ^b^ Goodness of fit tested by chi-square, all *P* values were more than 0.5 (degrees of freedom were 7); ^c^ LCR_50_, lethal concentration ratio at 50% response level (compared with LC_50_ of *O. hupensis* at 24 h exposure, LC_*O. h*_/LC_*B. a*_ or LC_*B. t.*_). If the 95% confidence interval of LCR_50_ includes 1, then the LC_50_s are not significantly different

### Field molluscicidal activity

**Fig. 4:**
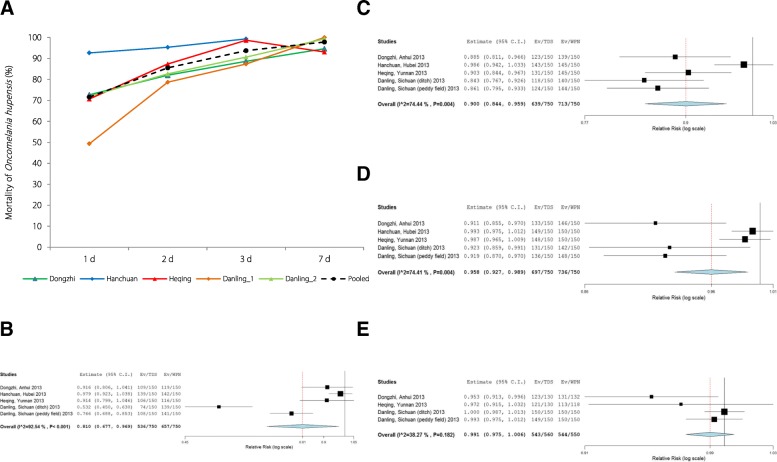
Molluscicidal effect of 4% TDS by immersion in the field. **a** Mortality of *Oncomelania hupensis* post-exposure to 4% TDS by immersion in the field. Danling_1, conducted in the ditch; Danling_2, conducted in the paddy field. **b–e** Comparison of *Oncomelania hupensis* mortalities (individual and pooled results) between TDS (2.5 g/m^3^) and WPN (2 g/m^3^) 1 (**b**), 2 (**c**), 3 (**d**) and 7 d (**e**) post-immersion in the field, studies performed 2011 to 2013. There is no significant difference in the pooled effects between TDS and WPN 7 d post-immersion (**e**), of which the relative risk values (TDS/WPN) for snail mortality was 0.991 (95% *CI*: 0.975–1.006)

Across all schistosomiasis-endemic regions selected for the field assessment of 4% TDS activity, the pooled mortalities of *O. hupensis* were 72% (95% *CI*: 56.7–86.6%), 86% (95% *CI*: 78.8–92.2%), 94% (95% *CI*: 89.6–97.7%) and 98% (95% *CI*: 95.7–99.9%) 1, 2, 3, and 7 d post-immersion, respectively (Fig. 4a and Additional file 2: Table S4) [18]. In comparison to the effects of 50% WPN treatment, the pooled effect of 4% TDS were lower than 50% WPN at 1, 2 and 3 d but not significantly different at 7 d post-immersion (Fig. 4b–e): the calculated 4% TDS/50% WPN RR values for snail mortality were 0.81 (95% *CI*: 0.677–0.969), 0.9 (95% *CI*: 0.844–0.959), 0.958 (95% *CI*: 0.927–0.989) and 0.991 (95% *CI*: 0.975–1.006) at 1, 2, 3 and 7 d post-immersion, respectively (Fig. 4b–e).

**Fig. 5:**
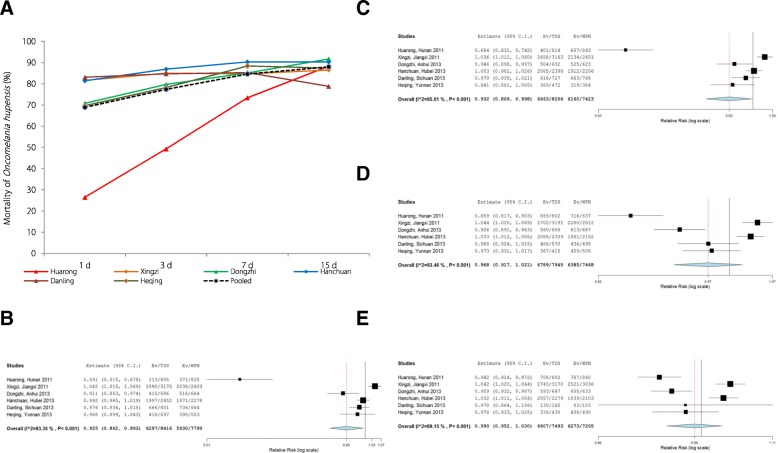
Molluscicidal effect of 4% TDS by spraying in the field. **a,** Mortalities of *Oncomelania hupensis* post-exposure by ground spraying of 4% TDS in the field. **b–e** Comparison of *Oncomelania hupensis* mortalities (individual and pooled results) between TDS (5 g/m^2^) and WPN (2 g/m^2^) 1 (**b**), 3 (**c**), 7 (**d**) and 15 d (**e**) post-spraying in the field, studies performed 2011 to 2013. There is no significant difference in the pooled effects between TDS and WPN at 7 d (**d**) and 15 d (**e**) post-spraying, of which the relative risk values (TDS/WPN) for snail mortality are 0.968 (95% *CI*: 0.917–1.021) at 7 d and 0.99 (95% *CI*: 0.952–1.030) at 15 d, respectively

Field conditions of the spraying trials are summarized in Additional file 2: Table S5. Across all regions, the pooled mortalities of *O. hupensis* were 69% (95% *CI*: 54.8–82.9%), 77% (95% *CI*: 69.4–85.4%), 85% (95% *CI*: 80.6–88.6%) and 88% (95 *CI*: 85.8–90.3%) 1, 3, 7 and 15 d post-spraying with 4% TDS, respectively (Fig. 5a and Additional file 2: Table S6). In comparison to 50% WPN treatment, the pooled effects of 4% TDS application were lower than those of WPN at 1 and 3 d but not significantly different at 7 and 15 d post-spraying (Fig. 5b–e): the pooled estimates of 4% TDS/50% WPN RR for snail mortality were 0.925 (95% *CI*: 0.862–0.993), 0.932 (95% *CI*: 0.869–0.998), 0.968 (95% *CI*: 0.917–1.021) and 0.99 (95% *CI*: 0.952–1.030) at 1, 3, 7 and 15 d post-spraying, respectively (Fig. 5b–e and Additional file 2: Table S6).

### Ecotoxicological activity of 4% TDS

Four percent TDS had moderate toxicity to quail (7 d LD_50_ > 60 mg/kg) and to shrimp (96 h LC_50_ = 6.28 mg/L; 95% *CI*: 3.53–11.2 mg/L), whereas its toxicity to zebrafish was high (96 h LC_50_ = 0.15 mg/L; 95% *CI*: 0.14–0.17 mg/L) (Table 2).

**Table 2:** Ecotoxicological tests of 4% TDS against *Coturnix japonica*, *Brachydanio rerio* and *Macrobrachium nipponense*

Species	Test	Duration and conditions	Toxicity
*Coturnix japonica*, aged 30 days, each weighing approximately 100 g, 5 males and 5 females for each group	Acute oral toxicity, repeated in triplicate	20–25 °C air temperature; 0, 6, 12, 24, 36, 60 mg/kg doses; a single dose by gavage; 7 days	1, 2, 3 and 7 d LD_50_ were above 60 mg/kg; moderate toxicity (50 < acute oral LD_50_ ≤ 500 mg/kg)
*Brachydanio rerio*, about 0.4 g in body weight and 3.5 cm in body length, 10 for each group	Acute toxicity, repeated in triplicate	20 °C water temperature; 0, 0.04, 0.1, 0.15, 0.2, 0.4 mg/L concentrations; semi-static, 96 h	24, 48, 72 and 96 h LC_50_ were 0.19 (0.16–0.22), 0.13 (0.12–0.14), 0.15 (0.14–0.17) and 0.15 (0.14–0.17) mg/L, respectively; high toxicity (0.1 < 96 h LC_50_ < 1.0 mg/L)
Adult *Macrobrachium nipponense*, about 2.0 g, 10 for each group	Acute toxicity, repeated in triplicate	20 °C water temperature; 0, 1, 2, 3, 5, 10 mg/L concentrations; semi-static, 96 h	24, 48, 72 and 96 h LC_50_ were > 10, 8.41 (5.54–12.8), 7.07 (4.96–10.1) and 6.28 (3.53–11.2) mg/L, respectively; moderate toxicity (1.0 < 96 h LC_50_ < 10 mg/L)

## Discussion

Currently, targeted mass delivery of anti-schistosomal drug therapy is the most common method used by national and regional schistosomiasis control programs across the world [1]. However, this approach has an important limitation: people at risk of infections who miss or decline treatment remain infected and continue to contribute to local transmission of *Schistosoma* parasites [19]. Multiyear experiences in mass treatment campaigns indicate that population participation declines over time, and further suggest that drug efficacy may be lower for those residents who continue to be infected after multiple rounds of treatment [20]. There is a need for additional practical interventions that can interrupt the process of parasite transmission [21]. In particular, there is a need for more selective and efficient molluscicides for control the snail intermediate hosts of this parasite [22].

In this regard, the present work describes the mollusciciding compound, 4% TDS, which is extracted from the plant *C. oleifera,* as a promising molluscicidal agent against *O. hupensis, B. alexandrina, and B. truncatus*, the snail intermediate hosts of the most common *Schistosoma* parasites of humans. This corresponds to the previously described molluscicidal activity of crude saponins extracted from *C. oleifera* seeds that has been shown for mollusk control in other pesticide test studies around the world [23–28]. Saponins are naturally occurring plant glycosides with a sugar moiety and an aglycone unit [29]. There is a high correlation between plants employed as fish poisons or soap substances and their molluscicidal activity [30]. Of note, it is known that the saponins from *Phytolacca dodecandra* or *Alternanthera philoxeroides* that are monodesmosidic (having a sugar moiety only at position C-3) possess a toxic activity, whereas bidesmosidic saponins (having a sugar moiety both at C-3 and C-28) are inactive [30–32].

For schistosomiasis control, niclosamide, the molluscicide used most frequently at present, has poor water solubility and has the disadvantage that it can stimulate *O. hupensis* snails to climb out of treated water to escape the chemical application, resulting in a reduction of the net molluscicidal effect [33]. Although TDS has a relatively lower lethal effect on snails, it, or other triterpenoid saponins, could be used as synergistic agents with currently available synthetic molluscicides.

The high molluscicidal activity of *P. dodecandra* is due to the presence of monodesmosidic saponin with an oleanolic acid glucoside base in the pericarp of the immature fruit of the plant *P. dodecandra* [34, 35]. Similarly, the high concentrations of saponins and flavonoids in the plants *Sesbania sesban*, *Euphorbia splendens*, *Cestrum purpureum,* and *Yucca filamentosa* “Marginata” contribute to their remarkable toxicity to *B. alexandrina* snails [36–39]. The toxicity of 4% TDS to snails may be due to their properties as protease inhibitors and their ability to interact with cholesterol, which produces insoluble substances that alter cellular activities, thereby resulting in cytotoxicity and death of the treated organisms [40].

The descending order of susceptibility of *O. hupensis*, *B. truncatus* and *B. alexandrina* snails to the toxic effects of 24 h exposure to 4% TDS is in parallel to previous results using *P. dodecandra* against *O. nosophora, B. truncatus,* and *B. glabrata* [41]. In comparable studies, *B. truncatus* was more susceptible than *B. alexandrina* to the toxic action of extracts from the plants *Euphorbia pseudocactus* and *E. helioscopia* [41]. It is therefore hypothesized that the variations in snail susceptibility to 4% TDS could be due to species differences in metabolic and physiological activities, including respiratory enzymes, protein and carbohydrate synthesis, and/or steroid sex hormone effects in treated snails [42, 43].

From the present work, the acute oral toxicity of 4% TDS to Japanese quail and shrimp was moderate. However, like niclosamide and *P. dodecandra*, 4% TDS can be lethal to zebrafish [44–46]. Because of the toxicity to aquatic species, 4% TDS should be cautiously used in water bodies.

Our study has several limitations. First, the activity of 4% TDS against *B. alexandrina* and *B. truncatus* were tested at only one time point (24 h), and further studies to assess the molluscicidal effect at long duration are required. Second, the field molluscicidal effect of 4% TDS against *B. alexandrina* and *B. truncatus* was not examined. This is because both *B. alexandrina* and *B. truncatus* are aquatic, and it is very difficult to perform a field assessment.

## Conclusions

The results of the present study demonstrate that 4% TDS is active against *O. hupensis*, *B. alexandrina* and *B. truncatus* under laboratory and field conditions, and it may be a candidate molluscicide of plant origin. Since snail control has been shown to be the most effective way to reduce schistosomiasis incidence and has been recommended to be included in global guidelines and national schistosomiasis control strategies [47, 48], it is therefore suggested that 4% TDS should be further tested for efficacy within operational research for schistosomiasis control in order to determine its ability to sustain the impact of chemotherapy in an integrated approach to controlling this highly prevalent parasitic disease [49].

## Additional files

Additional file 1: Multilingual abstracts in the five official working languages of the United Nations. (PDF 632 kb)

Additional file 2: **Table S1.** Mortality of *Oncomelania hupensis* by immersion test using 91.6% TDS. **Table S2.** Mortalities of *Oncomelania hupensis* by immersion with 4% TDS in the lab. **Table S3.** Mortalities of *Biomphalaria alexandrina* and *Bulinus truncatus* by immersion with 4% TDS in the lab. **Table S4.** Comparison of *Oncomelania hupensis* mortalities between TDS and WPN by immersion in the field. **Table S5.** Field conditions of the spraying test. **Table S6.** Comparison of *Oncomelania hupensis* mortalities between TDS and WPN by spraying in the field. (DOCX 45 kb)

## Abbreviations

CDC: Center for Disease Control and Prevention
*CI*: confidence interval
IUPAC: International Union of Pure and Applied Chemistry
JIPD: Jiangsu Institute of Parasitic Diseases
LC_50_: Median lethal concentration
LCR: Lethal concentration ratio
MOA: Ministry of Agriculture
PTS: Pentacyclic triterpenoid saponin
RR: Relative risk
TDS: Tea-seed distilled saponin
WHO: World Health Organization
WPN: Wettable powder of niclosamide ethanolamine salt
