# Corrections to: Molluscicidal effectiveness of Luo-Wei, a novel plant-derived molluscicide, against Oncomelania hupensis, Biomphalaria alexandrina and Bulinus truncatus

**DOI:** 10.1186/s40249-019-0548-2

**Published:** 2019-06-06

**Authors:** Tie-Wu Jia, Wei Wang, Le-Ping Sun, Shan Lv, Kun Yang, Neng-Min Zhang, Xi-Bao Huang, Jian-Bing Liu, Han-Cheng Liu, Rui-Hua Liu, Fathia A. Gawish, Mohamed R. Habib, Mohamed A. El-Emam, Charles H. King, Xiao-Nong Zhou

**Affiliations:** 1 National Institute of Parasitic Diseases, Chinese Center for Disease Control and Prevention, Shanghai, 200025 China; 2 Chinese Center for Tropical Diseases Research, Shanghai, 200025 China; 3 WHO Collaborating Centre for Tropical Diseases, Shanghai, 200025 China; 4 National Center for International Research on Tropical Diseases, Ministry of Science and Technology, Shanghai, 200025 China; 5 Key Laboratory of Parasite and Vector Biology, Ministry of Health, Shanghai, 200025 China; 6 Communicable Diseases Cluster, World Health Organization Regional Office for Africa (WHO/AFRO), PO Box 06, Brazzaville, Congo; 7 Key Laboratory of National Health Commission on Parasitic Disease Control and Prevention, Jiangsu Provincial Key Laboratory on Parasites and Vector Control Technology, Jiangsu Institute of Parasitic Diseases, Wuxi, 214064 China; 8 Hubei Jinhaichao Science & Technology Co.,Ltd, Wuhan, 430206 China; 9 Hubei Provincial Center for Disease Control and Prevention, Wuhan, 430079 China; 10 School of Chemistry and ChemicalEngineering, Wuhan Textile University, Wuhan, 430200 China; 11 Department of Medical Malacology, Theodor Bilharz Research Institute (TBRI), Imbaba, Giza, 12411 Egypt; 12 Center for Global Health and Diseases, Case Western Reserve University, Cleveland, OH USA; 13 Schistosomiasis Consortium for Operational Research and Evaluation, University of Georgia, Athens, GA USA

**Correction to: Infectious Diseases of Poverty (2019) 8:27**

**https://doi.org/10.1186/s40249-019-0535-7**

In the abstract of original publication of the article [1], 0.33 mg/L (24 h LC_50_ against *B. alexandrina*) should be replaced by 1.975 mg/L which was clearly showed in Table 1. We regret any confusion this error may have caused. The original publication has been corrected.
