# Supplementary material for: Molluscicidal effectiveness of Luo-Wei, a novel plant-derived molluscicide, against Oncomelania hupensis, Biomphalaria alexandrina and Bulinus truncatus
Source: Infect Dis Poverty. 2019 Mar 31;8:27. doi: 10.1186/s40249-019-0535-7 (PMC6480903; doi:10.1186/s40249-019-0535-7)
Supplement: Supplementary file 1 — Multilingual abstracts in the five official working languages of the United Nations. (PDF 632 kb) [file 40249_2019_535_MOESM1_ESM.pdf]

Translation of the abstract into the five official working languages of the United Nations

تأثير مبيد الرخويات لوه-وي، هو مبيد جديد مستخلص من النبات ، ضدحلزونOncomelania hupensis،حلزون ذاتُ السُّرَّتَينِ الإِسْكَنْدَرانِيَّةوحلزون Bulinus truncates

Bing -Bao Huang, Jian-Min Zhang, Xi-Ping Sun, Shan Lv, Kun Yang, Neng-Le ,Wei Wang, Wu Jia-Tie Emam, -Fathia A. Gawish, Mohamed R. Habib, Mohamed A. El ,Hua Liu-Cheng Liu, Rui-Liu, Han Nong Zhou-Charles H. King, Xiao

نُبذة

لمحة عامة:لقد ثبت أن التحكم في المضيفات الوسيطة الحلزونية هو نهج سريع وفعال لمقاطعة انتقال داء البلهارسيات. وقد أظهرت بعض المستخلصات النباتية نشاطًا واضحًا للمادة المولدة للديدان ، وقد تم تطوير مركب جديد يسمى لوه-وي ، يدعى أيضًا سابونين المقطر من بذور الشاي (TDS) ، استنادًا إلى الصابونين المستخرجة منكامي أوليفيراالبذور. هدفنا إلى اختبار نشاط الـ TDS بنسبة 4٪ TDS ضد الحلزون الوسيطة في الصين ومصر ، وتقييم سلامتها البيئية إلى الكائنات غير المستهدفة.

الطرق: في المعمل،حلزون Oncomelania hupensis،حلزون ذاتُ السُّرَّتَينِ الإِسْكَنْدَرانِيَّةوحلزون Bulinus truncatesتعرضت إلى 4 ٪ TDS ، وتركيز متوسط القاتلة ($_{50}$LC)قدرت في 24 و 48 و 72 ساعة. في هذا المجال ، تم تقييم الوفيات الحلزونية 1 و 2 و 3 و 7 بعد الغمر ب 2.5 غم / م^{3}4 ٪ TDS و 1 و 3 و 7 و 15 د بعد الرش مع 5 جم / م^{2}4% TDS بالإضافة إلى ذلك ، سمية حادة من 4 TDS ٪ إلى السمان الياباني (السمان الياباني)، دانيو مخطط (دانيو مخطط) و جمبري المياة العذبة (Macrobrachium nipponensis)تم تقييمهمن تقديرات $_{50}$LCأو الجرعة المميتة المتوسطة ($_{50}$LD).

النتيجة: في المعمل ، ال$_{50}$LCقيم 4٪ TDS لحلزون O. hupensisكانت 0.701 و 0.371 و 0.33 ملغم / لتر عند 24 و 48 و 72 ساعة على التوالي و 4٪ TDS أظهرت 0.33 ملغم / لتر 24 ساعة $_{50}$LCضدحلزون ذاتُ السُّرَّتَينِ الإِسْكَنْدَرانِيَّة، و 1.396 ملغم / لتر 24 ساعة $_{50}$LCضدحلزون B. truncatus. في جميع مناطق الدراسة ، والوفيات المجمعة منحلزون O. hupensكانت 72 ٪ ، 86 ٪ ، 94 ٪ و 98 ٪ في 1 ، 2 ، 3 و 7 د ، بعد غمر الحقل من المواد الصلبة الذائبة 4 ٪ بجرعة مقدارها 2.5 غرام / م^3، وكانت 69 ٪ ، 77 ٪ ، 85 ٪ و 88 ٪ في 1 و 3 و 7 و 15 د ، بعد رش الحقل في 5 جم / م^{2}على التوالى . 4 ٪ TDS كان سمية معتدلة إلى السمان الياباني (7 <g id = "12">50 > 60 d LD مجم / كجم) والروبيان (96 ساعة $_{50}$LC= 6.28 ملغم / لتر. 95٪3.53-11.2 ملغم / لتر) ، في حين أن سميتها إلى الزرد عالية (96 ساعة LC50= 0.15 ملغم / ل ؛ 95٪CI: 0.14 - 0.17 ملغم / لتر).

الاستنتاج: 4٪ TDS فعال ضد حلزون O. hupensis، وحلزون ذاتُ السُّرَّتَينِ الإِسْكَنْدَرانِيَّةوحلزون B. truncatusتحت ظروف المختبر والميدان ، ويمكن أن يكون مبيد الرشاد من أصل نباتي.

Translated from English version into Arabic by Mohamed Shawkat, proofread by Aalya Al-Beeshi, through

TRANSLATORS WITHOUT BORDERS

植物灭螺药"螺威"对湖北钉螺、亚历山大双脐螺和截形小泡螺的灭螺效果

贾铁武，汪伟，孙乐平，吕山，杨坤，张能敏，黄希宝，刘建兵，刘汉成，刘瑞华，Fathia A. Gawish，Mohamed R. Habib，Mohamed A. El-Emam，Charles H. King，周晓农

摘要

引言：控制中间宿主螺已被证实是一种快速、有效的阻断血吸虫病措施。一些植物提取物显示出具有杀螺活性。螺威，又称茶籽皂素（TDS），是一种从油茶科植物种子中提取的新型化合物。

本研究在中国和埃及测定 4% TDS 对血吸虫中间宿主螺的杀灭效果，评价其对非靶生物的环境安全性。
方法：在室内，湖北钉螺、亚历山大双脐螺和截形小泡螺分别暴露于 4% TDS，计算 24、48 和 72 h 药物半数致死浓度（LC_{50}）。在现场，统计 2.5 g/m^3 4% TDS 浸杀 1、2、3、7 d 和 5 g/m^2 4% TDS 喷洒 1、3、7、15 d 后的钉螺死亡率。观察 4% TDS 对日本鹌鹑、斑马鱼和淡水虾的急性毒性，统计 LC_{50} 或半数致死剂量（LD_{50}）。
结果：在室内，4% TDS 对湖北钉螺的 24、48 和 72 h LC_{50} 值分别为 0.701、0.371 和 0.33 mg/L，对亚历山大双脐螺的 24 h LC_{50} 值为 0.33 mg/L，对截形小泡螺的 24 h LC_{50} 值为 1.396 mg/L。现场采用 2.5 g/m^3 4% TDS 浸杀 1、2、3、7 d 后，湖北钉螺合并死亡率分别为 72%、86%、94%和 98%，采用 5 g/m^2 4% TDS 喷洒 1、3、7、15 d 后，湖北钉螺合并死亡率分别为 69%、77%、85%和 88%。4% TDS 对日本鹌鹑（7 d $LD_{50} > 60$ mg/kg）和淡水虾（96 h $LC_{50} = 6.28$ mg/L；95% *CI*：3.53–11.2 mg/L）为中等毒性，但对斑马鱼毒性较大（96 h $LC_{50} = 0.15$ mg/L；95% *CI*：0.14–0.17 mg/L）.
结论：4% TDS 在室内和现场对湖北钉螺、亚历山大双脐螺和截形小泡螺杀螺效果均较好，可作为一种候选植物灭螺药。

Translated from English version into Chinese by Wei Wang

Efficacité molluscicide du Luo-Wei, un nouveau molluscicide d'origine végétale, sur *Oncomelania hupensis*, *Biomphalaria alexandrina* et *Bulinus truncatus*

Tie-Wu Jia, Wei Wang, Le-Ping Sun, Shan Lv, Kun Yang, Neng-Min Zhang, Xi-Bao Huang, Jian-Bing Liu, Han-Cheng Liu, Rui-Hua Liu, Fathia A. Gawish, Mohamed R. Habib, Mohamed A. El-Emam, Charles H. King, Xiao-Nong Zhou

Résumé

Contexte: le contrôle des hôtes intermédiaires de type gastéropode s'est avéré un moyen rapide et efficace d'interrompre la transmission de la schistosomiase. Certains extraits végétaux présentent une activité molluscicide manifeste et un nouveau composé, appelé Luo-Wei ou TDS (pour «tea-seed distilled saponin»), a été développé à partir de saponines extraites de graines de *Camellia oleifera* (couramment appelées «graines de thé»). Cette étude visait à tester l'activité molluscicide de la TDS à 4 % contre des escargots servant d'hôtes intermédiaires aux schistosomes en Chine et en Égypte et à évaluer son innocuité environnementale pour les organismes non visés.
Méthodes: En laboratoire, *Oncomelania hupensis*, *Biomphalaria alexandrina* et *Bulinus truncatus* ont été exposés à de la TDS à 4 % et la concentration létale médiane (CL_{50}) a été estimée à 24, 48 et 72 heures. Sur le terrain, la mortalité des gastéropodes a été évaluée à 1, 2, 3 et 7 jours après submersion à raison de 2,5 g/m^3 de TDS à 4 % et à 1, 3, 7 et 15 jours après pulvérisation de 5 g/m^2 de TDS à 4 %. Par ailleurs, la toxicité aiguë de la TDS à 4 % sur la caille du Japon (*Coturnix coturnix japonica*), le poisson-zèbre (*Brachydanio rerio*) et la crevette d'eau douce du Japon (*Macrobrachium nipponensis*) a été évaluée sur la base d'estimations de la CL_{50} ou de la dose létale médiane (DL_{50}).
Résultats: En laboratoire, les CL_{50} de la TDS à 4 % atteignaient respectivement 0,701, 0,371 et 0,33 mg/l à 24, 48 et 72 h pour *O. hupensis*, 0,33 mg/l à 24 h pour *B. alexandrina* et 1,396 mg/l à 24 h pour *B. truncatus*. Dans l'ensemble des régions couvertes par l'étude, la mortalité regroupée d'*O. hupensis* s'élevait respectivement à 72 %, 86 %, 94 % et 98 % à 1, 2, 3 et 7 jours après submersion de terrains

avec de la TDS à 4 % à raison de 2,5 g/m^3, et 69 %, 77 %, 85 % et 88 % à 1, 3, 7 et 15 jours après pulvérisation sur le terrain à une dose de 5 g/m^2. La TDS à 4 % présentait une toxicité modérée sur la caille du Japon (DL_{50} à 7 jours > 60 mg/kg) et la crevette (CL_{50} à 96 h = 6,28 mg/l ; *IC* à 95 % de 3,53 à 11,2 mg/l), tandis que sa toxicité sur le poisson-zèbre était élevée (CL_{50} à 96 h = 0,15 mg/l ; *IC* à 95 % de 0,14 à 0,17 mg/l).

Conclusions: La TDS à 4 % est active contre *O. hupensis*, *B. alexandrina* et *B. truncatus* dans les conditions de laboratoire et sur le terrain et pourrait constituer un candidat molluscicide d'origine végétale.

Translated from English version into French by Eric Ragu, proofread by Suzanne Assenat, through

TRANSLATORS
WITHOUT BORDERS

Эффективность моллюскицидных свойств Ло-Вей, нового моллюскицида растительного происхождения, против *Oncomelania hupensis, Biomphalaria alexandrina* и *Bulinus truncates*

Ти-Ву Цзя, Вэй Ван, Ле-Пин Сунь, Шань Лю, Кунь Ян, Нэн-Минь Чжан, Си-Бао Хуан, Цзянь-Бин Лю, Хань-Чен Лю, Руи-Хуа Лю, Фатия А. Гавиш, Мохамед Р. Хабиб, Мохамед А. Эль-Эмам, Чарльз Х. Кинг, Сяо-Нонг Чжоу

Аннотация

Вводная информация: доказано, что контроль промежуточных хозяев улитки является быстрым и эффективным методом прерывания передачи шистосомоза. Некоторые растительные экстракты продемонстрировали очевидную моллюскицидную активность, и на основе сапонинов, экстрагированных из семян *Camellia oleifera*, было разработано новое соединение Ло-Вей, также называемое сапонином, дистиллированным из семян чайного дерева (TDS). Мы поставили задачу проверить моллюскицидную активность 4% TDS против промежуточных улиток-хозяев в Китае и Египте и оценить его экологическую безопасность для организмов, не являющихся мишенями.

Методы: *Oncomelania hupensis*, *Biomphalaria alexandrina* и *Bulinus truncatus* в лаборатории подвергались воздействию 4% TDS, а средняя смертельная концентрация (LC_{50}) была оценена через 24, 48 и 72 часа. В полевых условиях смертность улитки оценивалась через 1, 2, 3 и 7 дней после погружения с дозой 2,5 г/м^3 4% TDS и через 1, 3, 7 и 15 дней после распыления с дозой 5 г/м^2 4% TDS. Кроме того, была оценена острая токсичность 4% TDS для японских перепелов (*Coturnixcoturnix japonica*), рыбок данио (*Brachydonio rerio*) и пресноводных креветок (*Macrobrachium nipponensis*) с помощью оценок LC_{50} или средней смертельной дозы (LD_{50}).

Результаты: в лаборатории значения LC_{50} для 4% TDS для *O. hupensis* были 0,701, 0,371 и 0,33 мг/л через 24, 48 и 72 часа соответственно, и для 4% TDS были определены 0,33 мг/л LC_{50} через 24 часа против *B. alexandrina* и 1,36 мг/л LC_{50} через 24 ч против *B. truncatus*. Во всех регионах исследования суммарная смертность для *O. hupensis* составила 72%, 86%, 94% и 98% через 1, 2, 3 и 7 дней после полевого погружения в 4% TDS в дозе 2,5 г/м^3, и 69%, 77%, 85% и 88% через 1, 3, 7 и 15 дней после полевого распыления в дозе 5 г/м^2, соответственно. 4% TDS обладал умеренной токсичностью для японского перепела (7 дней LD_{50} > 60 мг/кг) и для креветок (96 ч

LC_{50} = 6,28 мг/л; 95% *ДИ*: 3,53–11,2 мг/л), тогда как его токсичность для рыбок данио была высока (96 ч LC_{50} = 0,15 мг/л; 95% *ДИ*: 0,14–0,17 мг/л).
Выводы: 4% TDS активен против *O. hupensis*, *B. alexandrina* и *B. truncatus* в лабораторных и полевых условиях, и он может быть рассмотрен как кандидат-моллюскицид растительного происхождения.

Translated from English version into Russian by Michael Orlov, proofread by Alexander Somin, through

TRANSLATORS WITHOUT BORDERS

Eficacia molusquicida de Luo-Wei, un nuevo molusquicida de origen vegetal, contra *Oncomelania hupensis, Biomphalaria alexandrina* y *Bulinus truncates*

Tie-Wu Jia, Wei Wang, Le-Ping Sun, Shan Lv, Kun Yang, Neng-Min Zhang, Xi-Bao Huang, Jian-Bing Liu, Han-Cheng Liu, Rui-Hua Liu, Fathia A. Gawish, Mohamed R. Habib, Mohamed A. El-Emam, Charles H. King, Xiao-Nong Zhou

Resumen
Contexto: el control de los caracoles que actúan como huéspedes intermediarios ha demostrado ser un método rápido y eficaz para interrumpir la transmisión de la esquistosomiasis. Algunos extractos de plantas han mostrado una actividad molusquicida obvia y un nuevo compuesto, Luo-Wei, también llamado saponina destilada de semillas de té (TDS, por sus siglas en inglés), fue desarrollado sobre la base de las saponinas extraídas de las semillas de *Camellia oleifera*. Nuestro objetivo fue examinar la actividad molusquicida de la TDS al 4 % contra los caracoles huéspedes intermediarios en China y Egipto, y evaluar su seguridad ambiental para los organismos no objetivo.
Metodología: en el laboratorio, *Oncomelania hupensis*, *Biomphalaria alexandrina* y *Bulinus truncatus* fueron expuestos a TDS al 4 %, y la concentración letal media (CL_{50}) fue estimada en 24, 48 y 72 hrs. En el campo, se evaluaron las mortalidades de caracoles a los 1, 2, 3 y 7 días de post-inmersión con 2,5 g/m^3 de TDS al 4 %, y a los 1, 3, 7 y 15 días de post-pulverización con 5 g/m^2 de TDS al 4 %. Además, la toxicidad aguda de la TDS al 4 % para la codorniz japonesa (*Coturnixcoturnix japonica*), el pez cebra (*Brachydonio rerio*) y las gambas de agua dulce (*Macrobrachium nipponensis*) fue evaluada mediante estimaciones de la CL_{50} o mediante la dosis media letal (DL_{50}).
Resultados: en el laboratorio, los valores de la CL_{50} de la TDS al 4 % para *O. hupensis* fueron 0,701, 0,371 y 0,33 mg/L a las 24, 48 y 72 hrs, respectivamente, y la TDS al 4 % mostró una CL_{50} a 0,33 mg/L a las 24 hrs contra *B. alexandrina,* y una CL_{50} a 1,396 mg/L a las 24 hrs contra *B. truncatus*. En todas las regiones estudiadas, las mortalidades agrupadas de *O. hupensis* fueron del 72 %, del 86 %, del 94 % y del 98 % a los 1, 2, 3 y 7 días, después de una inmersión de campo de TDS al 4 % a una dosis de 2,5 g/m^3, y fueron del 69 %, del 77 %, del 85 % y del 88 % a los 1, 3, 7 y 15 días, después de pulverizar el campo a 5 g/m^2, respectivamente. La TDS al 4 % tuvo una toxicidad moderada para la codorniz japonesa (7 días DL_{50} > 60 mg/kg) y para la gamba (96 hrs CL_{50}= 6,28 mg/L; 95 % *CI*: 3,53–11,2 mg/L), mientras que la toxicidad para el pez cebra fue elevada (96 hrs CL_{50}= 0,15 mg/L; 95% *CL*: 0,14–0,17 mg/L).

Conclusiones: la TDS al 4 % es activa contra *O. hupensis*, *B. alexandrina* y *B. truncatus* en condiciones de laboratorio y de campo, y puede ser un molusquicida candidato de origen vegetal.

Translated from English version into Spanish by Lia Sarra Felip, proofread by Mayra León, through

TRANSLATORS
WITHOUT BORDERS
