# Supplementary material for: Molluscicidal effectiveness of Luo-Wei, a novel plant-derived molluscicide, against Oncomelania hupensis, Biomphalaria alexandrina and Bulinus truncatus
Source: Infect Dis Poverty. 2019 Mar 31;8:27. doi: 10.1186/s40249-019-0535-7 (PMC6480903; doi:10.1186/s40249-019-0535-7)
Supplement: Supplementary file 2 — Table S1. Mortality of Oncomelania hupensis by immersion test using 91.6% TDS. Table S2. Mortalities of Oncomelania hupensis by immersion with 4% TDS in the lab. Table S3. Mortalities of Biomphalaria alexandrina and Bulinus truncatus by immersion with 4% TDS in the lab. Table S4. Comparison of Oncomelania hupensis mortalities between TDS and WPN by immersion in the field. Table S5. Field conditions of the spraying test. Table S6. Comparison of Oncomelania hupensis mortalities between TDS and WPN by spraying in the field. (DOCX 45 kb) [file 40249_2019_535_MOESM2_ESM.docx]

Additional file2: Table S1. Mortality of *Oncomelania hupensis* by immersion test using 91.6% TDS

| Drug concentration (mg/L) | Snail mortality (%, *n*/*N*) | | |
| --- | --- | --- | --- |
|  | 24 h | 48 h | 72 h |
| 0.500 | 100 (90/90) | 100 (90/90) | 100 (90/90) |
| 0.250 | 91.1 (82/90) | 96.7 (87/90) | 98.8 (89/90) |
| 0.125 | 36.7 (33/90) | 63.3 (57/90) | 71.1 (64/90) |
| 0.063 | 3.3 (3/90) | 8.3 (8/90) | 10.0 (9/90) |
| 0.031 | 0 (0/90) | 0 (0/90) | 0 (0/90) |
| Control | 0 (0/90) | 0 (0/90) | 0(0/90) |

Median lethal concentrations (LC_50_) by immersion for 24, 48, or 72 h were 0.142 (0.128–0.157), 0.110 (0.099–0.122), and 0.102 (0.092–0.112) mg/L, respectively.

Additional file2: Table S2. Mortalities of *Oncomelania hupensis* by immersion with 4% TDS in the lab

| Drug concentration (mg/L) |  | Snail mortality (%, *n*/*N*) | | | | |
| --- | --- | --- | --- | --- | --- | --- |
|  |  | 24 h |  | 48 h |  | 72 h |
| 10 |  | 100 (30/30) |  | 100 (30/30) |  | 100 (30/30) |
| 5 |  | 100 (30/30) |  | 100 (30/30) |  | 100 (30/30) |
| 2.5 |  | 93.33 (28/30) |  | 100 (30/30) |  | 100 (30/30) |
| 1.25 |  | 83.33 (25/30) |  | 96.67 (29/30) |  | 100 (30/30) |
| 0.63 |  | 53.33 (16/30) |  | 93.33 (28/30) |  | 96.67 (29/30) |
| 0.31 |  | 6.67 (2/30) |  | 33.33 (10/30) |  | 36.67 (11/30) |
| 0.16 |  | 0 (0/30) |  | 3.33 (1/30) |  | 6.67 (2/30) |
| 0.08 |  | 0 (0/30) |  | 0 (0/30) |  | 0 (0/30) |
| 0.04 |  | 0 (0/30) |  | 0 (0/30) |  | 0 (0/30) |
| Control |  | 0 (0/30) |  | 0 (0/30) |  | 0 (0/30) |

Additional file2: Table S3. Mortalities of *Biomphalaria alexandrina* and *Bulinus truncates* by immersion with 4% TDS in the lab

| Drug concentration (mg/L) | Snail mortality (%, *n*/*N*) | |
| --- | --- | --- |
|  | *Biomphalaria alexandrina* | *Bulinus truncates* |
| 0.75 | 0.0 (0/30) | 3.3 (1/30) |
| 1 | 3.3 (1/30) | 10.0 (3/30) |
| 1.25 | 6.7 (2/30) | 26.7 (8/30) |
| 1.5 | 16.7 (5/30) | 56.7 (17/30) |
| 1.75 | 26.7 (8/30) | 83.3 (25/30) |
| 2 | 46.7 (14/30) | 93.3 (28/30) |
| 2.25 | 63.3 (19/30) | 96.7 (29/30) |
| 2.5 | 83.3 (25/30) | 100 (30/30) |
| 2.75 | 93.3 (28/30) | 100 (30/30) |
| Control | 0 (0/30) | 0 (0/30) |

Additional file2: Table S4. Comparison of *Oncomelania hupensis* mortalities between TDS and WPN by immersion in the field

| County, year |  | Mortality of *Oncomelania hupensis* (%, *n*/*N*) | | | | | | | | | | | | | | |
| --- | --- | --- | --- | --- | --- | --- | --- | --- | --- | --- | --- | --- | --- | --- | --- | --- |
|  |  | 1 d | | |  | 2 d | | |  | 3 d | | |  | 7 d | | |
|  |  | TDS | WPN | *RR*[95%*CI*] |  | TDS | WPN | *RR*[95%*CI*] |  | TDS | WPN | *RR*[95%*CI*] |  | TDS | WPN | *RR*[95%*CI*] |
| Dongzhi, 2013 |  | 72.7 (109/150) | 79.3 (119/150) | 0.916 [0.806, 1.041] |  | 82.0 (123/150) | 92.7 (139/150) | 0.885 [0.811, 0.966] |  | 88.7 (133/150) | 97.3 (146/150) | 0.911 [0.855, 0.970] |  | 94.6 (123/130) | 99.2 (131/132) | 0.953 [0.913, 0.996] |
| Hanchuan 2013 |  | 92.7 (139/150) | 94.7 (142/150) | 0.979 [0.923, 1.038] |  | 95.3 (143/150) | 96.7 (145/150) | 0.986 [0.942, 1.033] |  | 99.3 (149/150) | 100 (150/150) | 0.993 [0.975, 1.012] |  | . | . | . |
| Heqing, 2013 |  | 70.7 (106/150) | 77.3 (116/150) | 0.914 [0.799, 1.046] |  | 87.3 (131/150) | 96.7 (145/150) | 0.903 [0.844, 0.967] |  | 98.7 (148/150) | 100 (150/150) | 0.987 [0.965, 1.009] |  | 93.1 (121/130) | 95.8 (113/118) | 0.972 [0.915, 1.032] |
| Danling_1, 2013 |  | 49.3 (74/150) | 92.7 (139/150) | 0.532 [0.450, 0.630] |  | 78.7 (118/150) | 93.3 (140/150) | 0.843 [0.767, 0.926] |  | 87.3 (131/150) | 94.7 (142/150) | 0.923 [0.859, 0.991] |  | 100 (150/150) | 100 (150/150) | 1.000 [0.987, 1.013] |
| Danling_2, 2013 |  | 72.0 (108/150) | 94.0 (141/150) | 0.766 [0.688, 0.853] |  | 82.7 (124/150) | 96.0 (144/150) | 0.861 [0.795, 0.933] |  | 90.7 (136/150) | 98.7 (148/150) | 0.919 [0.870, 0.970] |  | 99.3 (149/150) | 100 (150/150) | 0.993 [0.975, 1.012] |
| Combined |  | 71.5 (536/750) | 87.6 (657/750) | . |  | 85.2 (639/750) | 95.1 (713/750) | . |  | 92.9 (697/750) | 98.1 (736/750) | . |  | 97.0 (543/560) | 98.9 (544/550) | . |
| Pooled |  | 0.716 [0.567, 0.866] | 0.881 [0.820, 0.943] | 0.810 [0.677, 0.969] |  | 0.855 [0.788, 0.922] | 0.955 [0.940, 0.971] | 0.900 [0.844, 0.959] |  | 0.937 [0.896, 0.977] | 0.988 [0.977, 0.999] | 0.958 [0.927, 0.989] |  | 0.978 [0.957, 0.999] | 0.994 [0.986, 1.002] | 0.991 [0.975, 1.006] |

The dose concentration was 2.5 g/m^3^ for 4% TDS and 2 g/m^3^ for 50% WPN. Danling_1: conducted in the ditch; Danling_2: conducted in the paddy field. The individual *RR* (relative risk or risk ratio) and their pooled estimates were calculated by using the following formula: *RR* = snail mortality caused by TDS/snail mortality caused by WPN. Significant difference was determined only if one was not included in the confidence interval of *RR*.

Additional file2: Table S5. Field conditions of the spraying test.

| Study area | Month, Year | Environment | Spraying area | Elevation (m) | Vegetation | Air temp. (°C) | Precipitation (mm; of d) | Density of living snails (No. /0.1m^2^) | Natural mortality of snails (%) | Batch number of 4% TDS |
| --- | --- | --- | --- | --- | --- | --- | --- | --- | --- | --- |
| Huarong, Hunan | May to June, 2011 | Lake and marshland | 6000 m^2^ for each group | 27 | Grass and reed | 20–33 | 39 (6, 7 and 15 d) | 10.38 | 1.60 | 20110517 |
| Xingzi, Jiangxi | October, 2011 | Lake and marshland | 4500 m^2^ for each group | 21 | Grass | 15–29 | 0 | 61.72 | 2.52 | 20110517 |
| Dongzhi, Anhui | May to June, 2013 | River floodland | 3200 m^2^ for TDS (or WPN), 600m^2^ for control | 23 | Weeds | 22–31 | 65 (0, 1, 3, 8, 9, 10 and 12 d) | 11.11 | 0.00 | 20130301 |
| Hanchuan, Hubei | May, 2013 | River floodland | 2000 m^2^ for each group | 28 |  | 15–32 | 78 (3 and 10 d) | 10.43 | 2.54 | 20130303 |
| Danling, Sichuan | September to October， 2013 | Hill (orchard) | >6000 m^2^ for each group | 623 | Weeds and trees | 15–30 | 181 (1, 3, 9, 10, 12 and 13 d); 69 mm at 1 d and 57 mm at 3 d | 16.17 | ≤ 1.02 | 20130301 |
| Jiangning, Jiangsu | July to August, 2013 | Hill | 500 m^2^ for each group | 115 | Weeds | 25–39 | 13 (3 d) | 21.98 | 9.72 | 20130501 |
| Heqing, Yunnan | August to September, 2013 | Mountain | >6000 m^2^ for each group | 2201 | Weeds | 23–27 | 10 (5 d) | 11.66 | ≤ 0.67 | 20130303 |

^*^The snail mortalities were 12.0% (69/575), 11.8%, 11.9% and 12.4% 1, 3, 7 and 15 d post-spraying in the control group of the Jiangning county, respectively; and the area of each test section was less than that of the designed standard of the spraying test (3000 m^2^ for TDS or WPN, 600 m^2^ for blank control) due to the size limitation of terrain, so the data were excluded from the further analysis.

Additional file2: Table S6. Comparison of *Oncomelania hupensis* mortalities between TDS and WPN by spraying in the field

| County, year |  | Mortality of *Oncomelania hupensis* (%, *n*/*N*) | | | | | | | | | | | | | | | | | | |
| --- | --- | --- | --- | --- | --- | --- | --- | --- | --- | --- | --- | --- | --- | --- | --- | --- | --- | --- | --- | --- |
|  |  | 1 d | | | |  | | 3 d | | |  | 7 d | | | | |  | 15 d | | |
|  |  | TDS | WPN | *RR*[95%*CI*] |  | | TDS | | WPN | *RR*[95%*CI*] |  | TDS | WPN | *RR*[95%*CI*] |  | TDS | | | WPN | *RR*[95%*CI*] |
| Huarong, 2011 |  | 26.5  (213/805) | 44.8  (371/828) | 0.591 [0.515, 0.678] |  | | 49.3 (401/814) | | 72.0 (607/843) | 0.684 [0.631, 0.742] |  | 73.4  (589/802) | 85.5  (716/837) | 0.859 [0.817, 0.903] |  | 88.3  (708/802) | | | 93.7  (787/840) | 0.942 [0.914, 0.972] |
| Xingzi, 2011 |  | 81.6  (2590/3175) | 78.3  (2038/2603) | 1.042 [1.015, 1.069] |  | | 85.0 (2688/3163) | | 82.0 (2134/2601) | 1.036 [1.012, 1.060] |  | 84.7  (2702/3191) | 81.1  (2280/2812) | 1.044 [1.020, 1.069] |  | 86.5  (2743/3170) | | | 83.0  (2521/3036) | 1.042 [1.020, 1.064] |
| Dongzhi, 2013 |  | 70.8  (415/586) | 77.7  (516/664) | 0.911 [0.853, 0.974] |  | | 79.7 (504/632) | | 84.3 (525/623) | 0.946 [0.898, 0.997] |  | 85.1  (560/658) | 91.9  (613/667) | 0.926 [0.891, 0.963 |  | 91.7  (593/647) | | | 95.6  (605/633) | 0.959 [0.932, 0.987] |
| Hanchuan, 2013 |  | 81.4  (1997/2452) | 82.1  (1871/2278) | 0.992 [0.965, 1.019] |  | | 86.9 (2085/2398) | | 86.7 (1912/2206) | 1.003 [0.981, 1.026] |  | 90.3 (2085/2309) | 87.4  (1881/2152) | 1.033 [1.012, 1.055] |  | 90.3  (2057/2279) | | | 87.4  (1839/2103) | 1.032 [1.011, 1.054] |
| Danling, 2013 |  | 83.1  (666/801) | 85.2  (736/864) | 0.976 [0.936, 1.018 |  | | 84.7 (616/727) | | 86.6 (663/766) | 0.979 [0.939, 1.021] |  | 85.3  (486/570) | 88.1  (436/495) | 0.968 [0.924, 1.015] |  | 78.8  (130/165) | | | 80.6  (83/103) | 0.978 [0.864, 1.106] |
| Heqing, 2013 |  | 69.7  (416/597) | 72.0  (398/553) | 0.968 [0.899, 1.043] |  | | 78.2 (369/472) | | 83.1 (319/384) | 0.941 [0.881, 1.005] |  | 88.4  (367/415) | 90.9  (459/505) | 0.973 [0.931, 1.017] |  | 87.4  (376/430) | | | 89.4  (438/490) | 0.978 [0.933, 1.025] |
| Combined |  | 74.8  (6297/8416) | 76.1  (5930/7790) | . |  | | 81.2 (6663/8206) | | 83.0 (6160/7423) | . |  | 85.4 (6789/7945) | 85.5  (6385/7468) | . |  | 88.2  (6607/7493) | | | 87.1  (6273/7205) | . |
| Pooled |  | 0.689 [0.548，0.829] | 0.734  [0.645, 0.823] | 0.925 [0.862, 0.993] |  | | 0.774 [0.694, 0.854] | | 0.825 [0.788, 0.862] | 0.932 [0.869, 0.998] |  | 0.846 [0.806, 0.886] | 0.874 [0.839, 0.909] | 0.968 [0.917, 1.021] |  | 0.880 [0.858, 0.903] | | | 0.887 [0.841, 0.933] | 0.990 [0.952, 1.030] |

The dose concentration was 5 g/m^2^ for 4% TDS and 2 g/m^2^ for 50% WPN.
